# Identification of New *Helicobacter pylori* Subpopulations in Native Americans and Mestizos From Peru

**DOI:** 10.3389/fmicb.2020.601839

**Published:** 2020-12-14

**Authors:** Andrés Julián Gutiérrez-Escobar, Billie Velapatiño, Victor Borda, Charles S. Rabkin, Eduardo Tarazona-Santos, Lilia Cabrera, Jaime Cok, Catherine C. Hooper, Helena Jahuira-Arias, Phabiola Herrera, Mehwish Noureen, Difei Wang, Judith Romero-Gallo, Bao Tran, Richard M. Peek, Douglas E. Berg, Robert H. Gilman, M. Constanza Camargo

**Affiliations:** ^1^Division of Cancer Epidemiology and Genetics, National Cancer Institute, Rockville, MD, United States; ^2^Department of Pathology and Laboratory Medicine, Faculty of Medicine, The University of British Columbia, Vancouver, BC, Canada; ^3^Universidad Peruana Cayetano Heredia, Lima, Peru; ^4^Laboratório de Bioinformática, Laboratório Nacional de Computação Científica (LNCC/MCTIC), Petrópolis, Brazil; ^5^Department of Genetics, Ecology and Evolution, Institute of Biological Sciences, Federal University of Minas Gerais, Belo Horizonte, Brazil; ^6^Asociación Benéfica PRISMA, Lima, Peru; ^7^National Institute of Genetics, Mishima, Japan; ^8^Department of Genetics, Graduate School of Life Sciences, The Graduate University for Advanced Studies (SOKENDAI), Mishima, Japan; ^9^Division of Gastroenterology, Hepatology and Nutrition, Department of Medicine, Vanderbilt University Medical Center, Nashville, TN, United States; ^10^Frederick National Laboratory for Cancer Research, National Cancer Institute, Frederick, MD, United States; ^11^Department of Molecular Microbiology, Washington University School of Medicine in St. Louis, St. Louis, MO, United States; ^12^Department of International Health, Bloomberg School of Public Health, Johns Hopkins University, Baltimore, MD, United States

**Keywords:** Amerindians, ancestry, indigenous, hspAmerind, mestizo, Peru

## Abstract

Region-specific *Helicobacter pylori* subpopulations have been identified. It is proposed that the hspAmerind subpopulation is being displaced from the Americans by an hpEurope population following the conquest. Our study aimed to describe the genomes and methylomes of *H. pylori* isolates from distinct Peruvian communities: 23 strains collected from three groups of Native Americans (Asháninkas [ASHA, *n* = 9], Shimaas [SHIM, *n* = 5] from Amazonas, and Punos from the Andean highlands [PUNO, *n* = 9]) and 9 modern mestizos from Lima (LIM). Closed genomes and DNA modification calls were obtained using SMRT/PacBio sequencing. We performed evolutionary analyses and evaluated genomic/epigenomic differences among strain groups. We also evaluated human genome-wide data from 74 individuals from the selected Native communities (including the 23 *H. pylori* strains donors) to compare host and bacterial backgrounds. There were varying degrees of hspAmerind ancestry in all strains, ranging from 7% in LIM to 99% in SHIM. We identified three *H. pylori* subpopulations corresponding to each of the Native groups and a novel hspEuropePeru which evolved in the modern mestizos. The divergence of the indigenous *H. pylori* strains recapitulated the genetic structure of Native Americans. Phylogenetic profiling showed that Orthogroups in the indigenous strains seem to have evolved differentially toward epigenomic regulation and chromosome maintenance, whereas OGs in the modern mestizo (LIM) seem to have evolved toward virulence and adherence. The prevalence of *cagA*^+^/*vacA s1i1m1* genotype was similar across populations (*p* = 0.32): 89% in ASHA, 67% in PUNO, 56% in LIM and 40% in SHIM. Both *cagA* and *vacA* sequences showed that LIM strains were genetically differentiated (*p* < 0.001) as compared to indigenous strains. We identified 642 R-M systems with 39% of the associated genes located in the core genome. We found 692 methylation motifs, including 254 population-specific sequences not previously described. In Peru, hspAmerind is not extinct, with traces found even in a heavily admixed mestizo population. Notably, our study identified three new hspAmerind subpopulations, one per Native group; and a new subpopulation among mestizos that we named hspEuropePeru. This subpopulation seems to have more virulence-related elements than hspAmerind. Purifying selection driven by variable host immune response may have shaped the evolution of Peruvian subpopulations, potentially impacting disease outcomes.

## Introduction

*Helicobacter pylori* is an ancestral member of the gastric microbiota and remains as a common cause of stomach diseases, including cancer (Khalifa et al., 2010). *H. pylori* has accompanied humans in their migrations and mirrored their biogeographic distributions (Falush et al., 2003; Linz et al., 2007; Yamaoka, 2009). Native Americans diverged from East Asians ∼23,000 years ago (ya) and settled in Beringia (Moreno-Mayar et al., 2018a,b). They later migrated to the Americas ∼16,000 ya via the Bering Strait, and rapidly dispersed through this vast territory (Gravel et al., 2013; Waters, 2019), arriving in South America initially in the Amazonas, and then progressively moving to Andes and Pacific coastal regions ∼12,000–15,000 ya (Gravel et al., 2013; Mendes et al., 2020).

In Peru, the Native population was exceedingly affected by Inca rules that forced the population to migrate and admix in the Andes (O’Fallon and Fehren-Schmitz, 2011; Harris et al., 2018). Immigration tended to occur toward the Amazon and coast from the Andes (Harris et al., 2018) due to high-altitude (Bigham, 2016), although gene flow in the reverse direction was also observed (Rodriguez-Delfin et al., 2000; Sandoval et al., 2013). When the Spanish conquerors invaded the territory, they imposed the assimilation rule that hindered even more other population movements (Mumford, 2012). After Peruvian independence, a considerable proportion of the Native population admixed with the Spanish to generate the modern mestizo population (Lovell, 1992; Harris et al., 2018) that still preserve a strong Amerindian ancestry, unlike other Latin American mestizos (Pereira et al., 2012; Homburger et al., 2015). A recent report found a clear differentiation between central Andean and Amazonian Native populations (Borda et al., 2020).

Phylogenetically, modern *H. pylori* strains are divided in six major populations according to Multilocus Sequence Typing (MLST) analysis (based on seven housekeeping genes), including hspEAsia and hpEurope; hspEAsia is further subdivided into hspMaori and hspAmerind (Falush et al., 2003; Linz et al., 2007; Yamaoka, 2009). A seminal work by Kersulyte et al. (2010) found that Shimaa strains of *H. pylori* (hspAmerind) from the Amazonas are derived from hspEAsia, while Lima strains intermingled with hpEurope. It is proposed that hspAmerind have been progressively displaced by hpEurope due to selection for more fit genotypes (Dominguez-Bello et al., 2008; Maldonado-Contreras et al., 2013). More recently, Thorell et al. (2017) identified several *H. pylori* subpopulations that rapidly evolved in the Americas during the last 500 years, but their study had a limited number of strains from the central Andes and Amazonas, and did not include samples from Peruvian modern mestizos.

Our study aimed to describe the evolution and genetic structure of genomes and methylomes of *H. pylori* isolates from four distinct Peruvian populations, residing in the Andes, Amazonas and urban regions. We addressed two research questions: i) Do *H. pylori* strains isolated from modern mestizos have an Amerindian component? and ii) Does the genetic diversity of Peruvian *H. pylori* populations recapitulate the genetic structure of their host human populations?

## Materials and Methods

### Samples

#### Bacterial Samples

The genomes of 32 *H. pylori* strains from four geographically and culturally distinct regions were fully sequenced: 9 from Amazonian Asháninkas (ASHA), 5 from Amazonian Shimaas (SHIM), 9 from Andean Puno (PUNO), and 9 from modern mestizos in Lima (LIM). The strains were isolated from gastric material collected by swallowed string (ASHA and SHIM) or tissue biopsies (PUNO and LIM). LIM strains were isolated from patients with histologically confirmed non-atrophic gastritis. All individuals provided informed consent, and the study was approved by the Human Studies Committees of Johns Hopkins University (Baltimore, MD, United States), of AB Prisma and of Universidad Peruana Cayetano Heredia (Lima, Peru).

#### Human Samples

We used genotyping data from 74 Native American individuals from the same study populations (Borda et al., 2020). This set included the 23 individuals from whom the *H. pylori* strains were isolated. Briefly, these human populations correspond to an Aymara-speaking group (*n* = 16) collected near the Titicaca lake shore in Puno region, and two Amazonian groups that inhabit the Amazon Yunga area and belong to the Arawakan linguistic family (Asháninkas [*n* = 35] and Shimaa [*n* = 23]). DNA samples were genotyped using the Illumina Human Omni array 2.5M. A total of 1,927,769 autosomal single nucleotide polymorphisms (SNPs) passed quality control and were combined with 1000 Genomes populations (*n* = 250), resulting in a dataset with 324 individuals (Supplementary Table S1).

### SMRT/PacBio Sequencing, Genome Assembly, Annotation and Methylation Calls

Bacterial genomic DNA was extracted from the 32 *H. pylori* strains using the QIAamp DNA Minikit (QIAGEN, Hilden, Germany) and purified with QIAGEN Genomic-tip 100/G columns. The genomic DNA was sequenced using PacBio RSII at the NCI’s Frederick National Laboratory for Cancer Research following the manufacturer’s protocol to obtain complete and circular genome sequences. The *de novo* assembly of each genome was performed following the instructions of the hierarchical genome assembly process (HGAP), version 2.0 (Chin et al., 2013); a complete closed contig was obtained for each bacterial genome. The genomes were annotated in Prokka v1.12 software (Seemann, 2014). DNA methylation detection was performed using kinetic data from the sequencing process, and base modification detection was conducted using the protocol “RS_Modification_and_Motif_Analysis.1” from PacBio using SMRT software analysis (version 1.4.0). Each motif was analyzed on REBASE^1^ to find the associated restriction-modification (R-M) systems against the *H. pylori* gold standard DNA methylation motifs. Only methylation sites with a Phred-like quality value score of ≥50 were used for subsequent analysis. Plasmids were assembled independently into extrachromosomal elements.

### Bioinformatic Analyses

We employed 95 NCBI genomes from different *H. pylori* populations as references (Supplementary Table S2), including the Canadian hspAmerind strains Aklavik86 and Aklavik117 that were sequenced using 454 technology (Kersulyte et al., 2010). To characterize and compare the circularized bacterial chromosomes, the following analyses were conducted: (i) phylogenomics and population structure with and without external NCBI reference sequences; (ii) population genetics analyses of the two major virulence factors *vacA* and *cagA*; (iii) methylation motif frequencies and densities. In addition, we performed a phylogenetic analysis of plasmid sequences.

#### Phylogenomics Analysis

Average nucleotide identity using blast (ANIb) among genomes was calculated in Pyani v0.2.7 (Richter and Rossello-Mora, 2009) and the corresponding identity scores were hierarchical clustered using Morpheus^2^. Phylogenomics analysis was performed by applying two approaches. First, to understand the global distribution of the Peruvian strains, we reconstructed a SNP core phylogeny using KSNP v3.0 (Gardner et al., 2015; van Vliet and Kusters, 2015). Second, the study genomes were annotated with Prokka, and then the gff files were used to inferred the pan-genome with Roary v3.7.0 (Page et al., 2015) using a blast identity of 80% and the -s option (van Vliet, 2017). Then, the gene presence/absence matrix and the core genome alignment from the Roary outputs were used as template to obtain a local phylogenomics tree among strains using Fasttree 2 (Price et al., 2009). A root-to-tip analysis using the local phylogenomics tree was determined in TempEst (Rambaut et al., 2016). The divergence times were estimated using the LSD software (To et al., 2016), applying the bacterial mutation rates reported by Moodley et al. (2012), and the human ancestral divergence times reported by the Peruvian Human Genome Project (Harris et al., 2018). In addition, we built a phylogenomics tree to compare the hspEuropePeru with hpEuropeColombia and hpEuropeNicaragua. The phylogenies were visualized using iTOL v3 software (Letunic and Bork, 2016).

#### Bacterial and Human Ancestry Analyses

To determine the bacterial population structure, we obtained a genome-wide co-ancestry matrix extracted from the study genomes using *in silico* chromosome painting. Subsequently, we used the co-ancestry matrix as input to run fineSTRUCTURE v4 for 100,000 iterations to perform model-based clustering using a Markov chain Monte Carlo as previously described (Lawson et al., 2012; Yahara et al., 2013).

The human population structure was inferred using genetic clustering analysis on ∼1.9M SNPs. We also included data from 1000 Genomes populations (Genomes Project et al., 2015) representing the subcontinental groups: West African (GWD), West Central African (YRI), East African (LWK), South European (IBS), North European (CEU), South Asian (ITU), East Asian (CDX and JPT) and two admixed Latin American populations (CLM and PEL). We performed a linkage disequilibrium pruning with PLINK (Purcell et al., 2007) and running the genetic clustering with ADMIXTURE (Alexander et al., 2009). We used a cross-validation approach to identify the best K value for the clustering. We ran admixture for K values ranging from 4 to 8 ancestral clusters.

#### Bacterial Genome Consensus

To identify differences in the bacterial genome architecture, we applied the approach proposed by Tada et al. (2017). Briefly, orthologs gene clusters were obtained by the bidirectional best hit method and used to create a consensus genome template. The template was aligned against each complete genome from the study population and clustered based on blast similarity scores.

#### Bacterial Orthogroups Determination and Population Genetics of Major Virulence Factors

Orthogroups (OGs) among *H. pylori* strains were identified using OrthoFinder v2.2.3 (Emms and Kelly, 2015). We used the generated gene count matrix and the local phylogenomics tree to evaluate the gain and loss patterns of OGs across all the study strains. Briefly, we applied the gain-loss-duplication model with Poisson distribution and four discrete gamma categories using Count software (Csuros, 2010). We screened all OGs to identify differential gene families either gained or lost among the groups. We defined four categories: OGs lost in all hspEuropePeru strains, OGs gain in all hspEuropePeru strains, OGs lost in most hspAmerind strains, and OGs gain in most hspAmerind strains. Functional classification of the identified families was performed by BLAST + software against the conserved domain database (Marchler-Bauer et al., 2015).

We calculated the prevalence of *cagA* and *vacA* alleles and determined the number of haplotypes (H), haplotype diversity (Hd) and nucleotide diversity (Pi) for both genes using DnaSP v6 software (Rozas et al., 2017). Then, we estimated the genetic differentiation for *cagA* and *vacA* alleles among study strains by using the nearest neighbor statistic (Snn) test with gene flow (Nm) under 1000 iterations in DnaSP v6 software (Rozas et al., 2017). Neutrality deviations were calculated by the *z*-tests in Kumar et al. (2016). Natural selection intensification or relaxation of *cagA* alleles were obtained by using the RELAX algorithm (Wertheim et al., 2015). EPIYA and CRPIA motifs were detected according to the approach by Suzuki (Suzuki et al., 2011).

Finally, using point mutations (A2142G, A2143G, and A2142C) in 23S ribosomal RNA gene, we identified resistance to clarithromycin, a core antibiotic in *H. pylori* eradication therapy.

#### Phylogenetic Analysis of Plasmids

We compared our *H. pylori* plasmids with 44 NCBI complete plasmid sequences (Supplementary Table S3) with lengths ranging from 5 to 25 kilobases (Kb). Phylogenetic analysis of all plasmids was performed using KSNP v3.0 (Gardner et al., 2015; van Vliet and Kusters, 2015). The phylogeny was visualized using iTOL v3 software (Letunic and Bork, 2016).

#### Bacterial Methylation Analysis

The presence of R-M systems: R (I, II, and III), S and M genes in the core and accessory genomes were determined using Spine, AGEnt and ClustAGE (Ozer et al., 2014; Ozer, 2018). Densities per 1 kb for total N6-methyladenine (m6A), N4-methylcytosine (m4C) and other methylation types were estimated using an *in-house* customized bash script (available upon request). The frequencies of motifs with at least 80% methylation fractions (*n* = 621) in the sequenced *H. pylori* genomes were visualized as a Venn diagram using a web tool^3^. Subsequently, shared and novel methylation motifs were identified using REBASE database against the *H. pylori* gold standard (Roberts et al., 2015).

## Results

**FIGURE 1:**
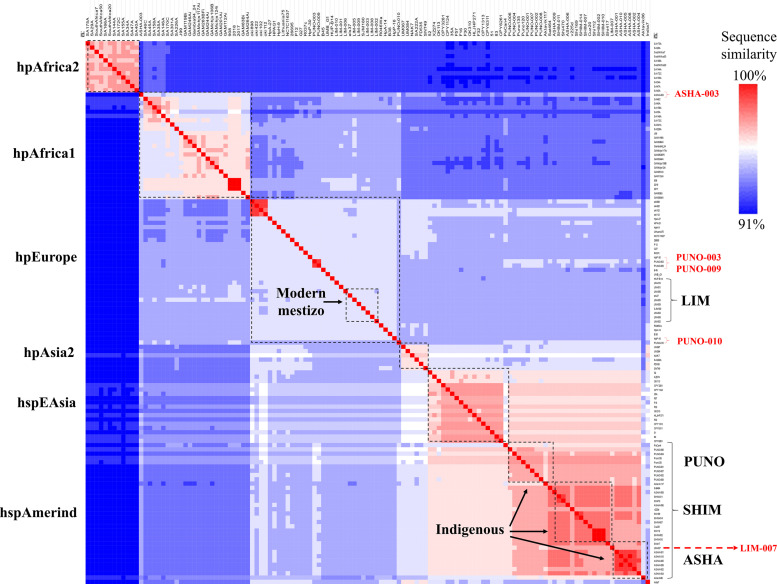
Hierarchical clustering analysis of ANIb values. Each line represents the similarity score of each *H. pylori* genome (32 study and 95 references). Left, ancestral *H. pylori* populations. Right, *H. pylori* study populations. Heavily admixed genomes (ASHA-003, PUNO-003, PUNO-009, PUNO-010, and LIM-007) are shown in red font.

Overall, the genome lengths and coding sequences ranged from 1.63 to 1.65 megabases (Mb) and from 1.59 to 1.62 Mb, respectively, similar to *H. pylori* genomes from other populations. The ANIb analysis revealed two major groups of strains: indigenous (ASHA, SHIM, and PUNO) and modern mestizo (LIM) (Figure 1). Although the two groups presented a high sequence similarity (ANIb ≥ 91–100%), the genomes from modern mestizo were more similar to those of hpEurope. We also identified that strains from the indigenous group (ASHA-003, PUNO-003, PUNO-009, and PUNO-010) shared components with the modern mestizo group, and one strain from the modern mestizo group (LIM-007) clustered with the indigenous (Figure 1). These five genomes represent heavily admixed strains.

**FIGURE 2:**
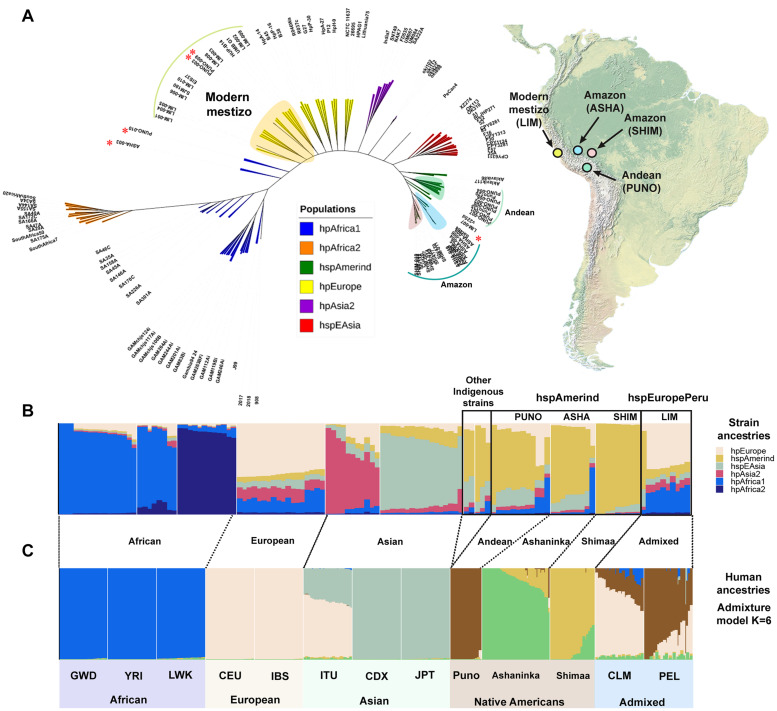
Phylogenomic relationships and population structures of *H. pylori* and human populations. **(A)** Global phylogenomic tree for 127 *H. pylori* strains. The tree was constructed from a total of 930,403 SNPs with a *K* value of 29 using KSNPV3.0. The shading blue (ASHA) and red (SHIM) are the strains obtained from the Amazon, the light green (PUNO) are the strains isolated from Puno in the Andes, and the light yellow (LIM) represent the strains isolated from Lima. The Peruvian map shows the regions where the samples were collected. The asterisks represent the heavily admixed strains (ASHA-003, PUNO-003, PUNO-009, PUNO-010, and LIM-007). **(B)** Ancestry profiles inferred using Chromopainter v2/fineSTRUCTURE for 127 *H. pylori* strains (32 study and 95 references; Supplementary Table S4). **(C)** ADMIXTURE results for 10 human populations from 1000 Genomes (*n* = 250) and Borda et al. (2020) (*n* = 74; Supplementary Table S1). We plot admixture results for the *K* value with the lowest cross validation error (*K* = 6; Supplementary Figure S2).

The core phylogenomic tree constructed from a total of 930,403 SNPs with a K value of 29 showed that all indigenous strains were grouped into independent clusters that define the hspAmerind subpopulation located next to the hspEAsia population. The modern mestizo strains were located near to the hpEurope population (Figure 2A). The divergence time estimates for all indigenous strains (ASHA, SHIM, and PUNO) was ∼13,512–9,000 ya. The ancestry analysis confirmed that the indigenous group was composed by three hspAmerind-like subclades. The hspAmerind ancestry component varied by population: ASHA ranged from 13 to 86%, SHIM from 90 to 99%, and PUNO from 15 to 68%. Except for LIM-007, with 64% of hspAmerind ancestry, all other LIM strains had less than 15% hspAmerind ancestry and were classified as hspEuropePeru. This new subpopulation is different from hpEuropeNicaragua and hpEuropeColombia (Supplementary Figure S1). Interestingly, we observed that ASHA-003 had a 50% of hpAfrica1 ancestry, while PUNO-003 and PUNO-009, 46 and 47% of hpEurope ancestry, respectively (Supplementary Table S4). The human ancestry analysis also showed that the Central Andean population (Puno) is differentiated from the Amazon populations (Shimaa and Asháninkas) (Figures 2B,C). Supplementary Figure S2 shows the evolution of the human clusters; *K* = 4 identified the four continental populations (Africa, Europe, Asian, and Native American), while *K* = 8 further discriminate the three African populations as Native American groups have higher genetic drift levels than Africans. A cross-validation approach identified *K* = 6 as the best value for the clustering.

**FIGURE 3:**
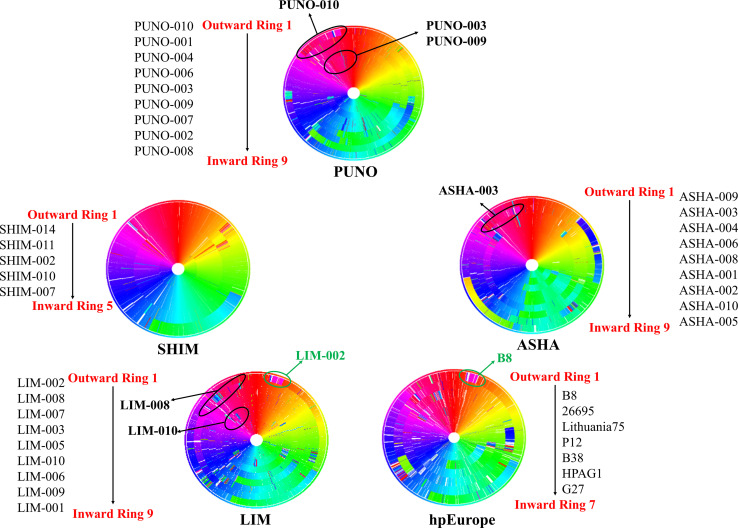
Genomic consensus of the study populations and hpEurope references. These circular views were obtained using the method developed by Tada et al. (2017), which creates a consensus genome that is used as a template for alignment. Each ring represents one complete genome and each block in the ring represents a genomic region. Different colors represent the genes according to their genomic position in the consensus. The shifts in the color represent the rearrangements. The outermost ring is the distant genome from the consensus. The names alongside each circle indicate the genomes going from outward to inward direction. The indicated areas (black circles) in the ASHA (ring 2) and PUNO (rings 1, 5, and 6) genomes show the regions in which these genomes are similar to the LIM (rings 2 and 6). The green circles indicate the similarities between the LIM genomes and the hpEurope references. The genomes ASHA-003, PUNO-003, PUNO-009, and PUNO-010 had an inversion from 10 o’clock to 11 o’clock which was not observed in SHIM genomes.

The bacterial consensus analysis using complete genomes showed that the indigenous group (ASHA, SHIM, and PUNO) have a homogeneous genomic architecture except for a few small insertions, transpositions and deletions. Likewise, although the indigenous group and modern mestizo strains share some rearrangements, the latter appeared to be more similar to the hpEurope strains (Figure 3).

The pangenome analysis indicates the core (i.e., >99% sequence similarity) genome contained 1,238 genes. Likewise, we also identified soft (i.e., 95 to 99%), shell (i.e., 15 to 94%), and cloud (i.e., <15%) genes (18, 375, and 346, respectively) that integrated a 1,996 pangenome. The genes were further clustered into 1,819 OGs that accounted for 99.4% of the genes. Table 1 shows the OGs by the evolutionary patterns of gene gain and loss based on a phylogenetic profiling. In general, OGs in the indigenous strains seem to have evolved differentially toward epigenomic regulation and chromosome maintenance, whereas OGs in the modern mestizo (LIM) seem to have evolved toward virulence, adherence, and phage protection. Supplementary Figure S3 shows examples of the gain and loss of OGs among study strains.

**TABLE 1:** Gain and loss patterns of orthogroup in hspAmerind and hspEuropePeru strains.

Orthogroup*	**CDD accession code**	**Protein family**	**Function**	Evolutionary patterns of gene gain and loss**
OG0001415	pfam04556	*Dpn*II restriction endonuclease	They recognize the double-stranded unmethylated sequence GATC and cleave before G-1	All hspEuropePeru strains lost these genes
OG0001416	pfam13146	TRL-like	No function reported	
OG0001694	COG1196	Smc chromosome segregation ATPase	Bacterial chromosome segregation	
OG0001428	cl35079	Alanyl-tRNA synthetase	Translational fidelity and proteome homeostasis	
OG0001607		Hypothetical		All hspEuropePeru strains gain these genes
OG0001669		Hypothetical		
OG0001670		Hypothetical		
OG0001693		Hypothetical		
OG0001701		Hypothetical		
OG0001702		Hypothetical		
OG0001703		Hypothetical		
OG0001490	COG2253	AbiEii toxin	Bacterial immunity	Most hspAmerind lost these genes
OG0001430	cl39651	SabA N-terminal extracellular adhesion domain	Binding to Lewis(B) and sialyl-Lewis(X) antigens on epithelial cells	
OG0001475	cl09751	R.Pab1 restriction endonuclease	Restriction endonuclease DNA glycosylase	
OG0001477	COG4772	Iron(III) dicitrate transport FecA_2	Inorganic ion transport and metabolism	
OG0001558	COG1598	HicB nuclease of the RNAse H fold	Bacterial immunity	
OG0001523	COG2084	MmsB 3-hydroxyisobutyrate dehydrogenase	Valine, leucine and isoleucine degradation (KEGG pathway: hpw00280)	
OG0001508	cd19078	AKR AKR13C family of aldo-keto reductase	Stomach acid adaptation	
OG0001523		Hypothetical		
OG0001768		Hypothetical		
OG0001649		Hypothetical		
OG0001650		Hypothetical		
OG0001424	BlastP	HrgC	Possible toxin (bacterial immunity)	Most hspAmerind gain these genes
OG0001481	cl00222	Lysozyme_like domain	Hydrolysis of beta-1,4-linked polysaccharides	
OG0001482	cl09780	*Hae*III restriction GG^CC	A restriction endonuclease that recognizes and cleaves GG^CC pattern	
OG0001484	cl00184	CAS_like Calvaminic acid synthase	No function reported	
OG0001497	cl00083	HNH nucleases	No function reported	
OG0001556	cl37069	SMS chromosome segregation protein	Structural maintenance of chromosomes	
OG0001616	cl01747	SMI1/KNR4	Potential bacterial immunity	
OG0001617	pfam14441	A nuclease HNH/ENDO VII	Potential bacterial immunity	
OG0001420		Hypothetical		
OG0001513		Hypothetical		
OG0001545		Hypothetical		
OG0001555		Hypothetical		

*As defined by Orthofinder. The protein sequence of one member of each orthofinder cluster was used as a query in the database of conserved domains (CDD). Functions were defined based on the output of CDDs. **Representative examples are presented in Supplementary Figure S3.

The prevalence of the combination *cagA*^+^/*vacA s1i1m1* genotype was similar across populations (*p* = 0.32; Fischer’s exact): 89% in ASHA, 67% in PUNO, and 56% in LIM and 40% in SHIM (Supplementary Table S5). For both *cagA*^+^ and *vacA s1i1m1* genes, Pi was considerably low, with overall values of 0.083 for *cagA* and 0.089 for *vacA*. Both genes also showed high Hd, with population averages of 0.994 for *cagA* and 0.987 for *vacA*. For both *cagA* and *vacA*, the *snn* tests showed that LIM was genetically differentiated (*p* < 0.001) as compared to the indigenous strains. Gene flow was also low (Nm 0.70 for *cagA* and 1.74 for *vacA*) and indicated a limited genetic exchange among populations. The *z*-tests showed signals of balancing and purifying selection for both virulence factors (Table 2). We found that the test RELAX showed significant results for selection intensification (*k* = 1.15, *p* = 0.037, and LR = 4.37). As expected, all study strains contain the EPIYA-ABC motif (Supplementary Figure S4). We found the AM-CRPIA motif in 70% (16/23) of indigenous strains and the W-CRPIA motif in all mestizo strains.

**TABLE 2:** Population and evolutionary statistics for *cagA* and *vacA*. Gdiv, genetic diversity estimators; n, number of sequences; S, number of segregating sites; h, number of haplotypes; Hd, haplotype diversity; K, average number of differences; Pi, Nucleotide diversity.

	**Populations**	**Gdiv**						**Gdif**	**GF**	***Z*-test**
		
		***n***	**S**	**h**	**Hd**	***K***	**Pi**	**Snn**	**FsT/Nm**	**dN < dS**
**CagA**	LIM	6	246	6	1.000	107.27	0.038			
	PUNO	7	473	7	1.000	223.90	0.079			
	SHIM	5	240	4	0.900	116.20	0.041			
	ASHA	9	455	8	0.972	171.78	0.060			
	Total	27	768	25	0.994	236.51	0.083	0.70***	0.41615/0.70	7.838***
**VacA**	LIM	9	769	9	1.000	331.36	0.088			
	PUNO	9	841	9	1.000	355.03	0.094			
	SHIM	5	470	4	0.900	269.90	0.071			
	ASHA	9	491	6	0.833	151.11	0.040			
	Total	32	1073	29	0.987	337.74	0.089	0.62***	0.22309/1.74	11.595***

All the estimators were presented per gene. Gdif/GF, Genetic differentiation and genetic flow. The genetic heterogeneity was detected using the Snn test from haplotype frequencies under 1000 permutations. Genetic flow was detected using the FsT from haplotype diversity under the permutation of 1000 repetitions. The Nm parameter was estimated from the FsT test. The deviation of the neutral model of molecular evolution was calculated using the *z*-tests. *Z*-test: Codon-based test of neutrality for analysis averaging over all sequence pairs. The probability of rejecting the null hypothesis of strict-neutrality (dN = dS) is shown. Values of *P* less than 0.05 are considered significant at the 5% level and are highlighted (***p < 0.001). The test statistics: dN > dS positive selection; dN < dS purifying selection are shown. dS and dN are the numbers of synonymous and non-synonymous substitutions per site, respectively. The variance of the difference was computed using the bootstrap method (1000 replicates). Analyses were conducted using the Nei-Gojobori method and all positions containing gaps and missing data were eliminated. Gdiv, Gdif, and GF were performed using DnaSP v 5.10. The *Z* tests was performed in Mega V 7.1. ^∗∗∗^*P* < 0.001.

Regarding the cag pathogenicity island (cagpai), the average number of genes among the 27 *H. pylori* genomes with this genomic region was 33 for all indigenous populations combined and 38 for mestizos. DNA alignments of cagpai sequence showed that SHIM strains were very similar with a small difference in length, with no insertions, inversions, transpositions or deletions. ASHA and PUNO shared similar patterns of inversions and transpositions, but ASHA showed more variability in sequence length than PUNO. On the other hand, LIM showed the highest inter and intra sequence complexity with many insertions, deletions and transpositions (Supplementary Figure S5).

We determined that one (LIM-003; A2142G mutation) of the 9 LIM strains could be classified as clarithromycin resistant, while no mutations were found in the 23 indigenous *H. pylori* strains.

**FIGURE 4:**
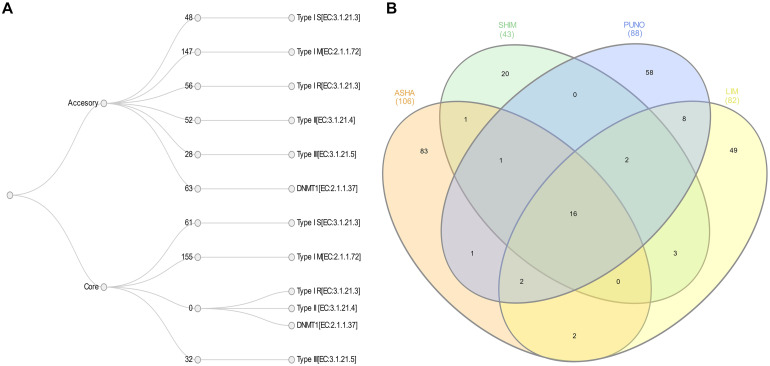
**(A)** Restriction-modification systems content in core and accessory *H. pylori* genomes from indigenous (9 ASHA, 5 SHIM, and 9 PUNO) and mestizos (9 LIM). **(B)** Venn diagram of methylation motifs with at least 80% methylation fractions.

We identified a total of 642 R-M systems among the four populations with 39% of them located in the core genome and 61% in the accessory. The average number of R-M genes by population was 174, the lowest found in SHIM strains with 108, and the highest in LIM strains with 198. Type I M, type I S and type III R genes were present in both the core and accessory genomes. The average number of core genes of type I M, type I S, and type III R genes were 39, 15, and 8 in the core genome and 37, 12, and 7 in the accessory genome, respectively (Figure 4A). In contrast, we found that the type I R, type II R, and *DNMT1* genes were only present in the accessory genome; including duplications, the 32 strains had averages of 14, 13, and 17 genes of these types, respectively.

The average methylation densities for m6A motifs per kb were 42 for ASHA and 39 for the other SHIM, PUNO and LIM; whereas for m4C motifs were 6 for ASHA, 8 for PUNO and LIM and 10 for SHIM. We found a total of 692 methylation motifs (with ≥80% of methylated sites) in the 32 genomes, including 254 novel motif sequences. Only 16 motifs were present in all four populations (at least one strain). There were no significant differences in the average motif number by population (22 for ASHA, 17 for SHIM, 21 for PUNO, and 22 for LIM) or in the average number of unique motifs (9 in ASHA, 4 in SHIM, 6 in PUNO, and 5 in LIM) (Figures 4A,B).

Among 32 genomes, we identified five (15.7%) plasmids, ASHA-003, ASHA-006, LIM-002, LIM-003, and LIM-005. Considering study and NCBI (*n* = 44) plasmids, lengths and GC content ranged from 5 to 25 Kb and from 31.7 to 37.5%, respectively. All plasmid sequences shared 2,916 common SNPs, 561 of which were homoplastic. The SNP phylogeny revealed five major clades showing a mixture of *H. pylori* populations and subpopulations as follows: (i) hpAsia2/hspEAsia, (ii) hpAsia2, (iii) hspEAsia/hspAmerind, (iv) hpEurope/hpAsia2/hpAfrica2, and (v) hspAfrica1/hpEurope/HpAsia2 (Supplementary Table S4 and Supplementary Figure S6).

## Discussion

Based on MLST analysis, it is proposed that the hspAmerind subpopulation has been progressively displaced by the hpEurope population. We used a cutting-edge sequencing technology to describe the genomic and epigenomic microevolution of *H. pylori* isolates from Peruvian populations. We found that hspAmerind is present in Native Americans and even traces are observed in modern mestizos.

Our findings suggest that hspAmerind-like populations in Peru may have evolved by a founder effect, following the divergence between the human Central-Southern Andean and Amazon populations (Borda et al., 2020). We found that the *H. pylori* divergence estimates dates follow along with the human divergence timing dates (Rodriguez-Delfin et al., 2000; Rothhammer and Dillehay, 2009; Gravel et al., 2013; Sandoval et al., 2013; Harris et al., 2018), and that the three hspAmerind-like populations had followed the genetic structure of their corresponding Amerindian human populations. It is of interest to determine the minimum rate of evolution at which new *H. pylori* subpopulations emerge and its determinants.

The genome consensus and ancestry analyses showed that indigenous and modern mestizo strains shared not only some rearrangements but also ∼15% of common ancestry, suggesting that modern mestizo strains still retain a significant hspAmerind component. However, modern mestizo strains have transitioned toward an hpEurope-like subpopulation and have been subject to a more aggressive genome erosion than the indigenous strains. Our results confirmed that the indigenous group is composed by a set of three well-differentiated hspAmerind-like subpopulations (SHIM, ASHA, and PUNO) that support the idea that hspAmerind-like subpopulations are present even in urbanized cities (Puno) that were affected by the Spanish conquerors. Complementary, modern mestizo strains were assigned to the hpEurope population, suggesting that their demography was recently shaped due to the introduction of new genetic material after the conquest in early 1,500s (Dufour and Piperata, 2004; Homburger et al., 2015; Adhikari et al., 2016; Mendes et al., 2020). Thus, following the nomenclature convention proposed by Thorell et al. (2017), we named this new subpopulation hspEuropePeru.

Our data suggest that hspEuropePeru and hspAmerind-like subpopulations seem to have evolved different gene content repertoires with potential phenotypic consequences. The following examples illustrate previous supporting evidence for the importance of some of the identified OGs. Kojima and Kobayashi (2015) found that in hspAmerind strains, the *pab1* restriction endonuclease gene was replaced by the *hrgC* (encoding a potential toxin) before its divergence from the hspEastAsia. In agreement, we found that the hspAmerind-like subpopulations (including LIM-007) have a copy of the *hrgC*, while the hspEuropePeru subpopulation have a copy of the *pab1*. Remarkably, our data suggest that the *pab1* was recently acquired by the hspEuropePeru subpopulation as a result of the human admixture with the conquerors. We also observed that hspEuropePeru contains the AbiEii system that is involved in phage-infected cell abortion (Dy et al., 2014), the *fecA2* that is associated with iron metabolism (van Vliet, 2017), and the *tonB* nickel transporter gene that is important for the stomach colonization (Schauer et al., 2007). In contrast, we found that most hspAmerind have lost *sabA* that encodes a sialic acid-binding adhesion protein with an important function on *H. pylori* infection chronicity (Mahdavi et al., 2002), contributing to its virulence. Future studies are warranted to replicate our findings and further characterize and understand the potential selective advantage of the modern mestizo *H. pylori* strains.

Related to major virulence factors, we found that the hspEuropePeru had a Western CagA type, whereas the hspAmerind-like carried a less virulent Amerindian type. As an expansion of our previous work (Kersulyte et al., 2010), we showed that *cagA* has diversified into a set of well-differentiated alleles that may represent a response against host immune challenge. It seems that the hspAmerind-like subpopulations optimized their co-evolutionary balance with the indigenous host. On the other hand, hspEuropePeru is still under the evolutionary arms race with its host following a red queen pattern (Morran et al., 2011; Defraine et al., 2018) that may have induced the evolution of a more aggressive bacterial phenotype.

*Helicobacter pylori* has a massive R-M system repertoire (Krebes et al., 2014) that continues to be revealed by technological advances. Our results suggest that ∼10% of the genome encodes R-M systems. Notably, the type I and II R-M systems were located exclusively in the accessory genome, supporting the hypothesis that restriction enzymes may be part of a bacterial defensive network that contribute to lineage homogenization (Sneppen et al., 2015; Oliveira et al., 2016). Likewise, we found that overall, 1/3 of methylation motifs were population-specific with no previous report in REBASE. There is not a universal motif across our 32 methylomes; only 2.4% (15 motifs) were present in all four population at least in one strain. The high diversity observed in population-specific methylation motifs suggests a reduction of gene transfer among populations with different motifs set, but also points toward the existence of specific gene fluxes among populations with the same motif repertoire (Oliveira et al., 2016). This diversity implies that each population was subject to a very intense diversifying population-specific selection that shaped its methylomes contributing to the geographic differentiation observed among the bacterial subpopulations in Peru (Xu et al., 2000; Kobayashi, 2001; Vale et al., 2009). Functional validation of identified R-M systems is critical as some methylation motifs may be spurious.

Plasmids are key extrachromosomal elements that not only provide novel functions to bacterial cells (i.e., antibiotic resistance), but also, they can increase the mutation rate and fitness (Hulter et al., 2017). Unlike the bacterial core-genome, the phylogenetic tree of plasmids is characterized by the presence of clades with mixed populations. Although, we identified a set of core SNPs shared by all plasmids suggesting a common ancestor, the lack of phylogeographic discrimination might be a consequence of the limited number of homoplastic traits that emerged independently in the mixed clades by convergent evolution. Plasmid diversity may reflect deep roots of evolutionary history of *H. pylori*. However, for a full characterization of plasmid diversity and evolution of *H. pylori*, large-scale studies in diverse populations are needed.

In conclusion, our study describes the evolution of hspAmerind and hspEuropePeru subpopulations from a larger ecological perspective, sampling individuals from different isolated communities. Both hspAmerind-like and hspEuropePeru subpopulations shared a significant common ancestry. We identified three hspAmerind-like subpopulations in Peru, one of them identified in Puno, a colonial city heavily impacted by the Spanish conquest. Also, we found that hspEuropePeru locally evolved in the modern mestizo. All subpopulations presented a very diverse methylome characterized by its population-specific motif repertoire. While our study adds to the understanding of the *H. pylori* admixture, further studies should address this phenomenon in other human communities with complex and recent migration patterns. We speculate that immune selection and lineage homogenization due to the bacterial R-M defensive system may be the force forging the evolution of *H. pylori* subpopulations not only in Peru but also in the Americas, and might help explaining the variable clinical outcomes associated with chronic *H. pylori* infection.

## Data Availability Statement

The datasets presented in this study can be found in online repositories. The names of the repository/repositories and accession number(s) can be found in the article/Supplementary Material.

## Author Contributions

AJG-E, ET-S, DEB, RHG, and MCC: study concept and design. BV, VB, ET-S, LC, JC, CCH, HJ-A, PH, JR-G, BT, RP, and DEB: acquisition of data. AJG-E, VB, CSR, ET-S, MN, DW, DEB, RG, and MCC: analysis and interpretation of data. AJG-E, MH, DW, and VB: statistics and bioinformatics. AJG-E, CSR, DW, and MCC: drafting of the manuscript. CSR, RHG, and MCC: obtained funding. RHG and MCC: study supervision. All authors: critical revision of the manuscript for important intellectual content. All authors contributed to the article and approved the submitted version.

## Conflict of Interest

The authors declare that the research was conducted in the absence of any commercial or financial relationships that could be construed as a potential conflict of interest.

## Footnotes

1 http://rebase.neb.com/rebase/rebase.html

2 https://software.broadinstitute.org/morpheus/

3 http://bioinformatics.psb.ugent.be/webtools/Venn
