# Supplementary material for: Identification of New Helicobacter pylori Subpopulations in Native Americans and Mestizos From Peru
Source: Front Microbiol. 2020 Dec 14;11:601839. doi: 10.3389/fmicb.2020.601839 (PMC7767971; doi:10.3389/fmicb.2020.601839)
Supplement: Supplementary file 1 [file Presentation_1.PPTX]

## Slide 1
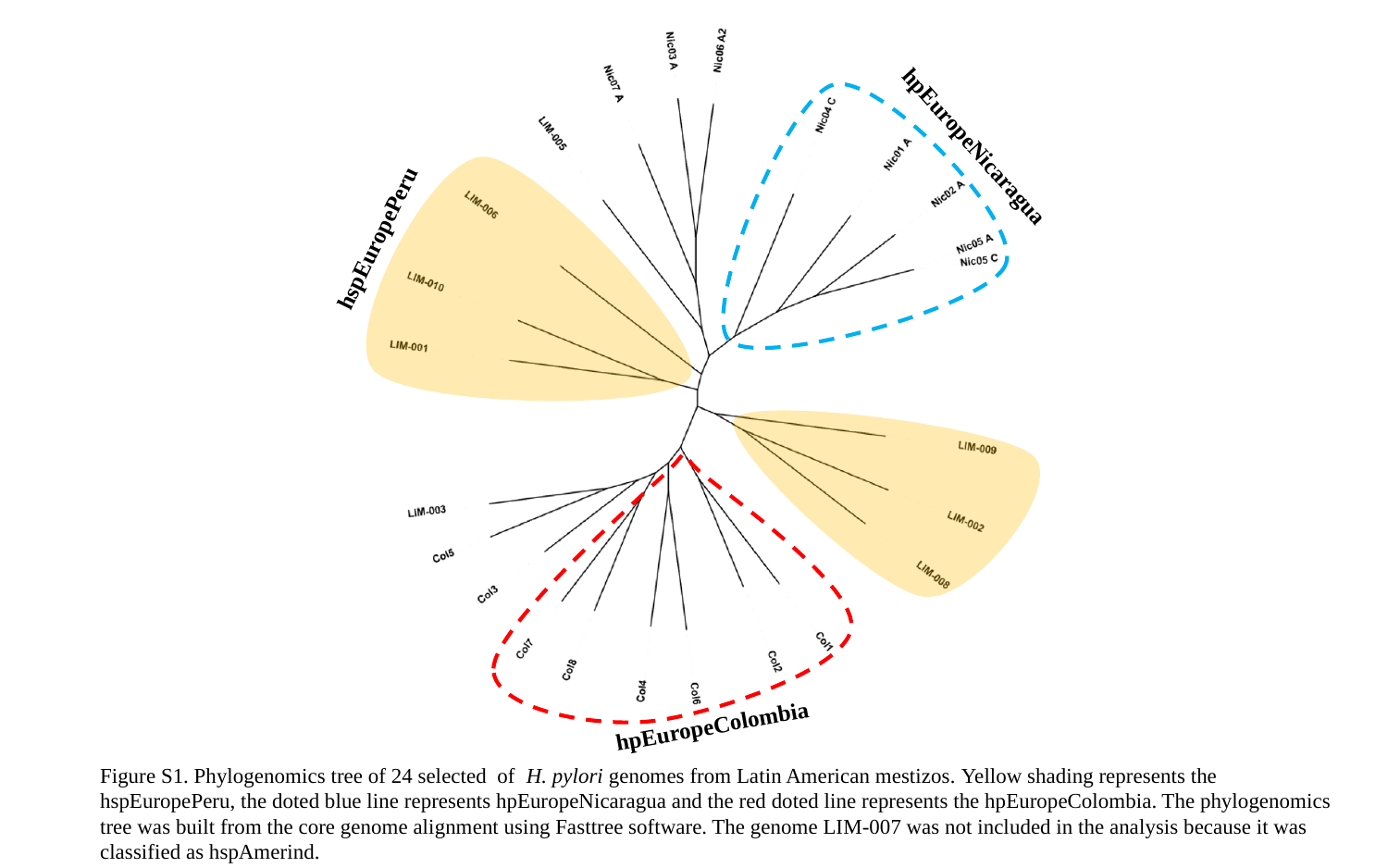

hpEuropeNicaragua
hspEuropePeru
hpEuropeColombia
Figure S1. Phylogenomics tree of 24 selected of H. pylori genomes from Latin American mestizos. Yellow shading represents the hspEuropePeru, the doted blue line represents hpEuropeNicaragua and the red doted line represents the hpEuropeColombia. The phylogenomics tree was built from the core genome alignment using Fasttree software. The genome LIM-007 was not included in the analysis because it was classified as hspAmerind.

## Slide 2
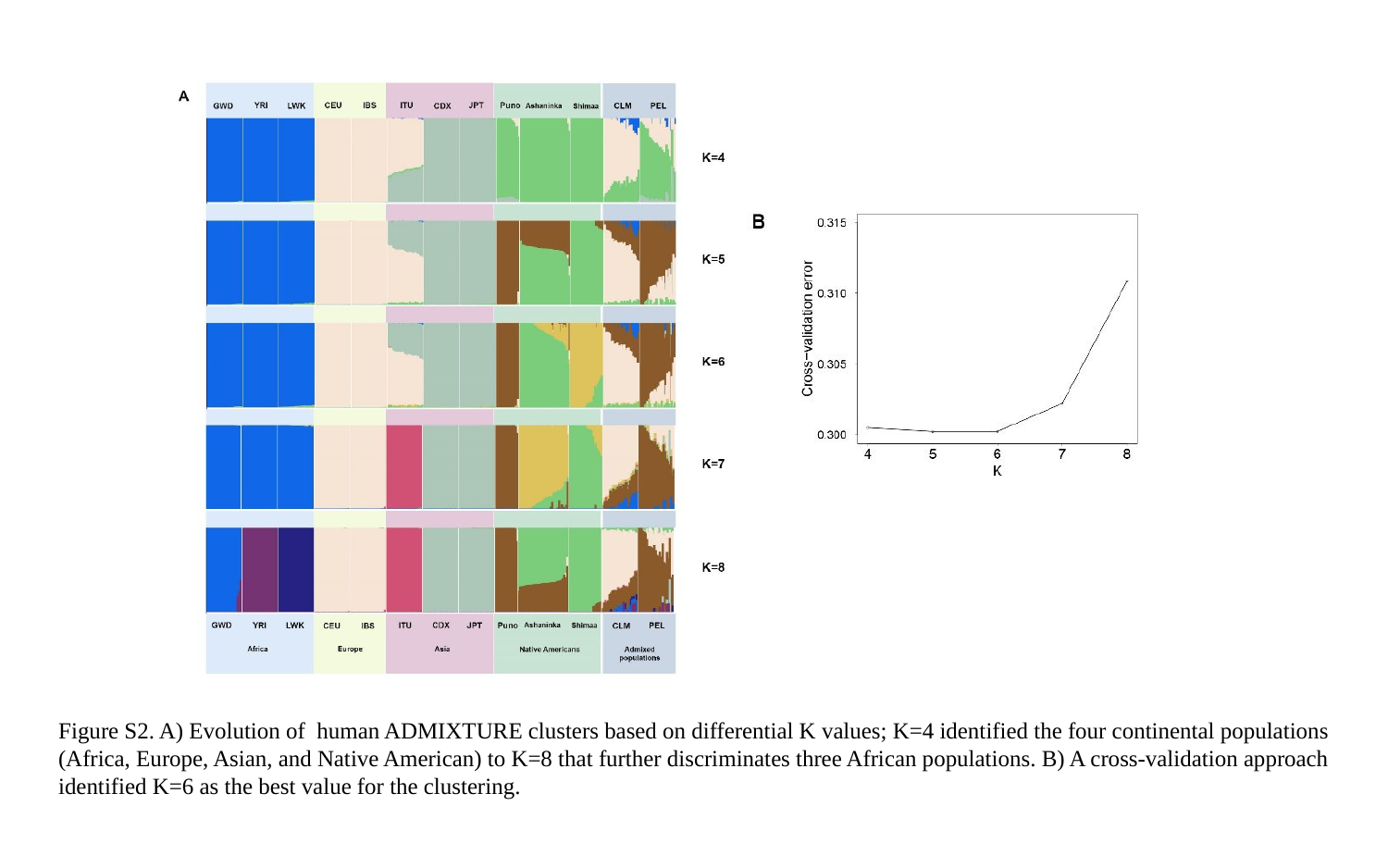

Figure S2. A) Evolution of human ADMIXTURE clusters based on differential K values; K=4 identified the four continental populations (Africa, Europe, Asian, and Native American) to K=8 that further discriminates three African populations. B) A cross-validation approach identified K=6 as the best value for the clustering.

## Slide 3
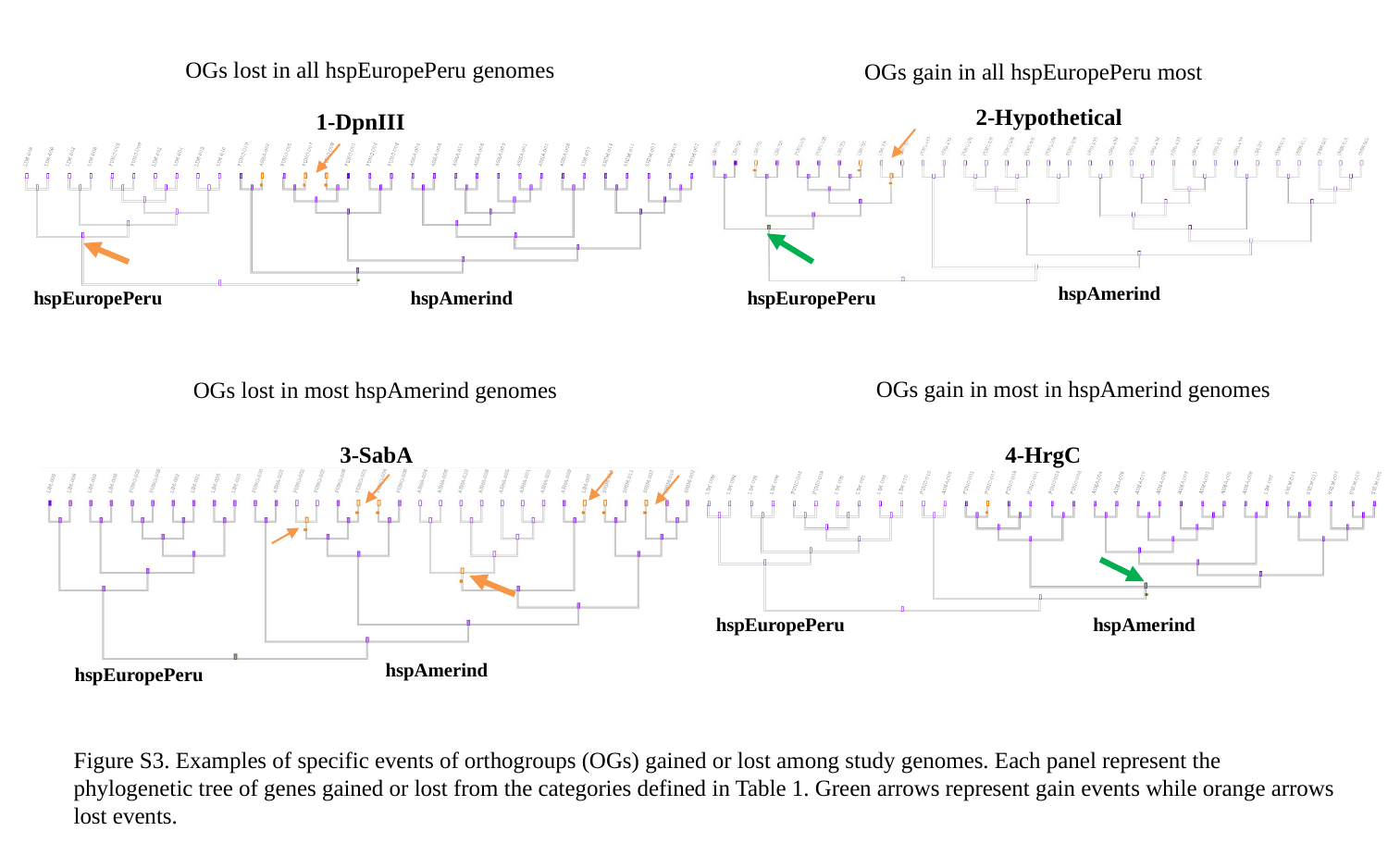

OGs lost in all hspEuropePeru genomes
OGs gain in all hspEuropePeru most
2-Hypothetical
1-DpnIII
hspAmerind
hspEuropePeru
hspAmerind
hspEuropePeru
OGs gain in most in hspAmerind genomes
OGs lost in most hspAmerind genomes
4-HrgC
3-SabA
hspEuropePeru
hspAmerind
hspAmerind
hspEuropePeru
Figure S3. Examples of specific events of orthogroups (OGs) gained or lost among study genomes. Each panel represent the phylogenetic tree of genes gained or lost from the categories defined in Table 1. Green arrows represent gain events while orange arrows lost events.

## Slide 4
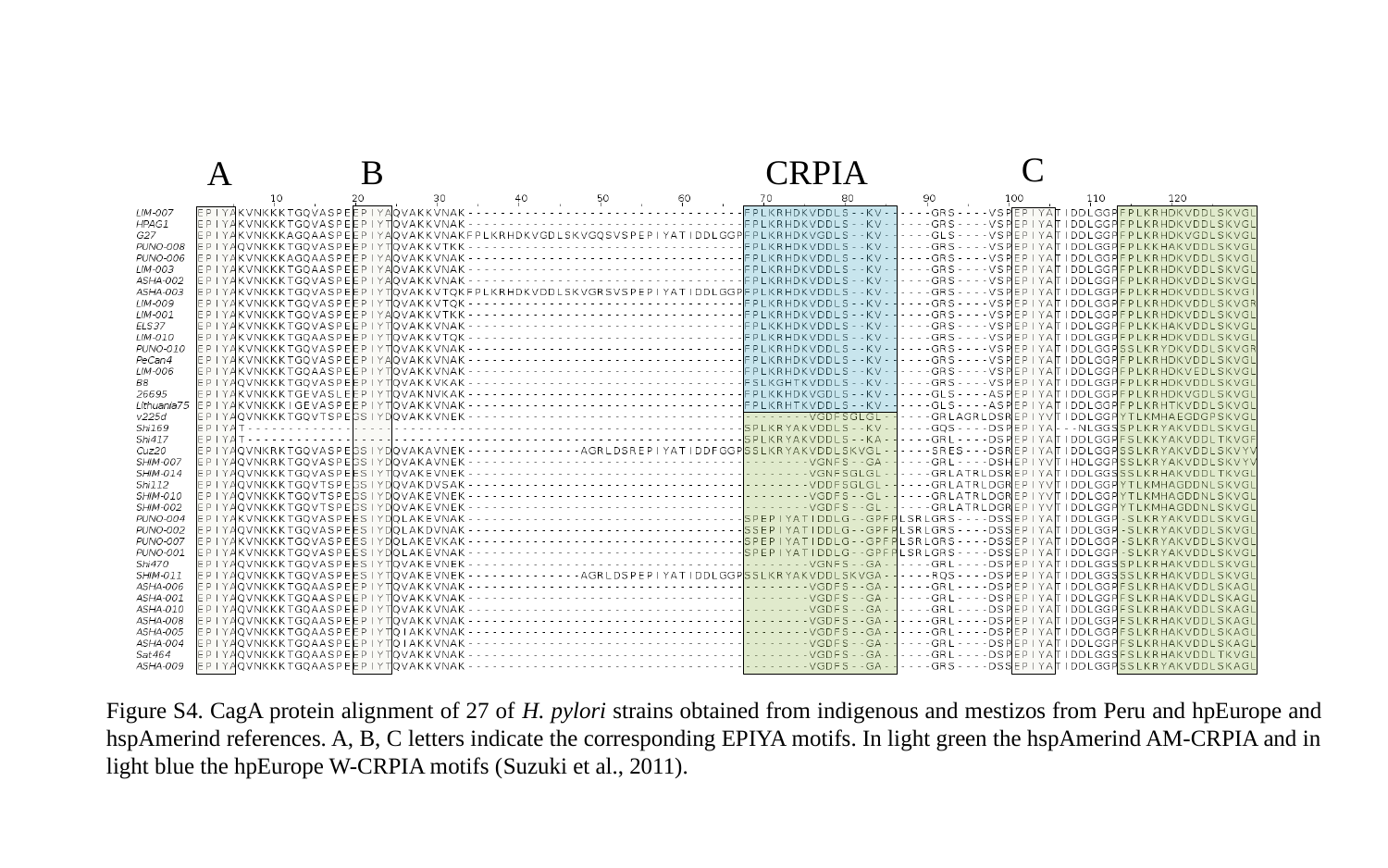

C
CRPIA
B
A
Figure S4. CagA protein alignment of 27 of H. pylori strains obtained from indigenous and mestizos from Peru and hpEurope and hspAmerind references. A, B, C letters indicate the corresponding EPIYA motifs. In light green the hspAmerind AM-CRPIA and in light blue the hpEurope W-CRPIA motifs (Suzuki et al., 2011).

## Slide 5
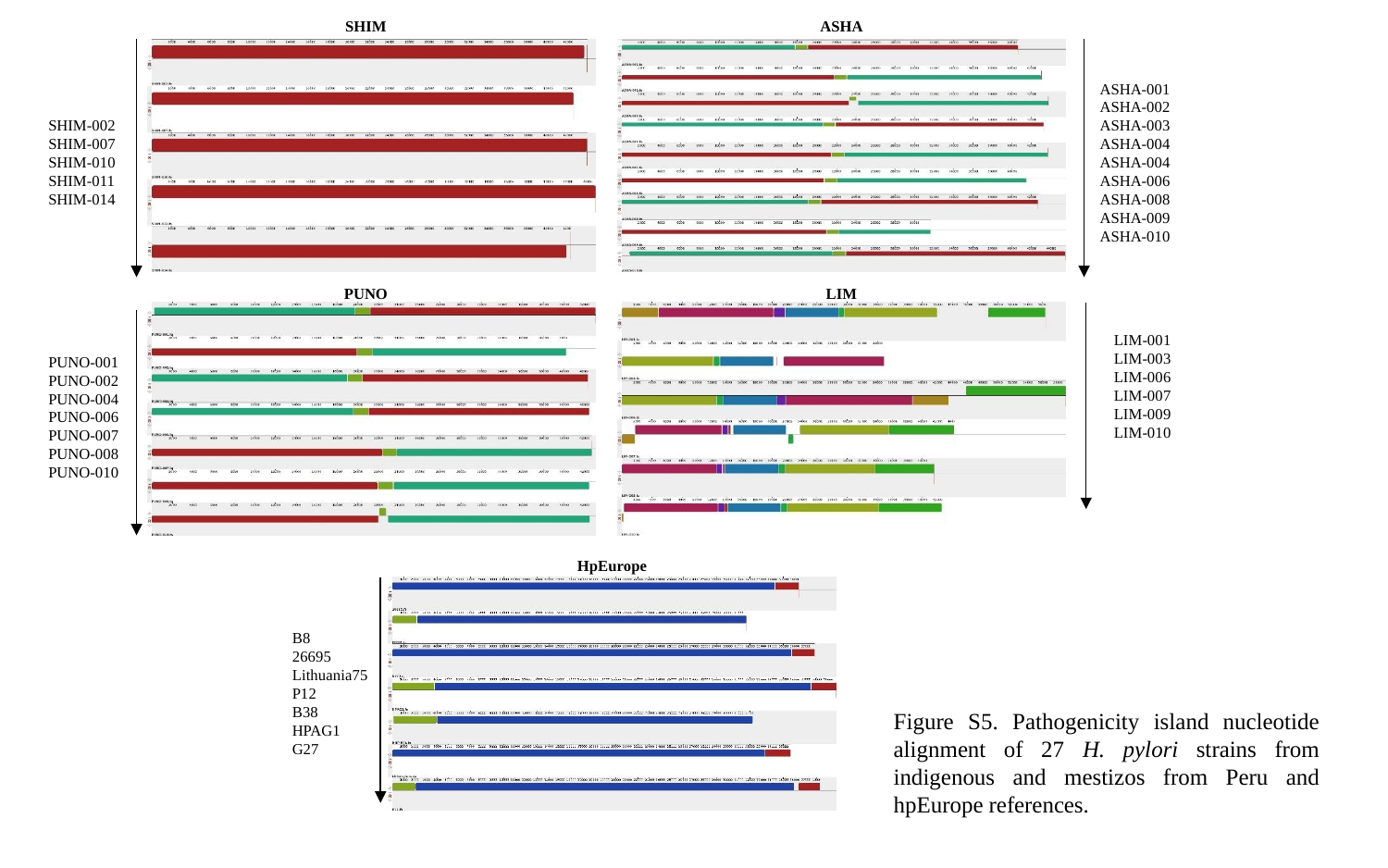

SHIM
ASHA
ASHA-001
ASHA-002
ASHA-003
ASHA-004
ASHA-004
ASHA-006
ASHA-008
ASHA-009
ASHA-010
SHIM-002
SHIM-007
SHIM-010
SHIM-011
SHIM-014
PUNO
LIM
LIM-001
LIM-003
LIM-006
LIM-007
LIM-009
LIM-010
PUNO-001
PUNO-002
PUNO-004
PUNO-006
PUNO-007
PUNO-008
PUNO-010
HpEurope
B8
26695
Lithuania75
P12
B38
HPAG1
G27
Figure S5. Pathogenicity island nucleotide alignment of 27 H. pylori strains from indigenous and mestizos from Peru and hpEurope references.

## Slide 6
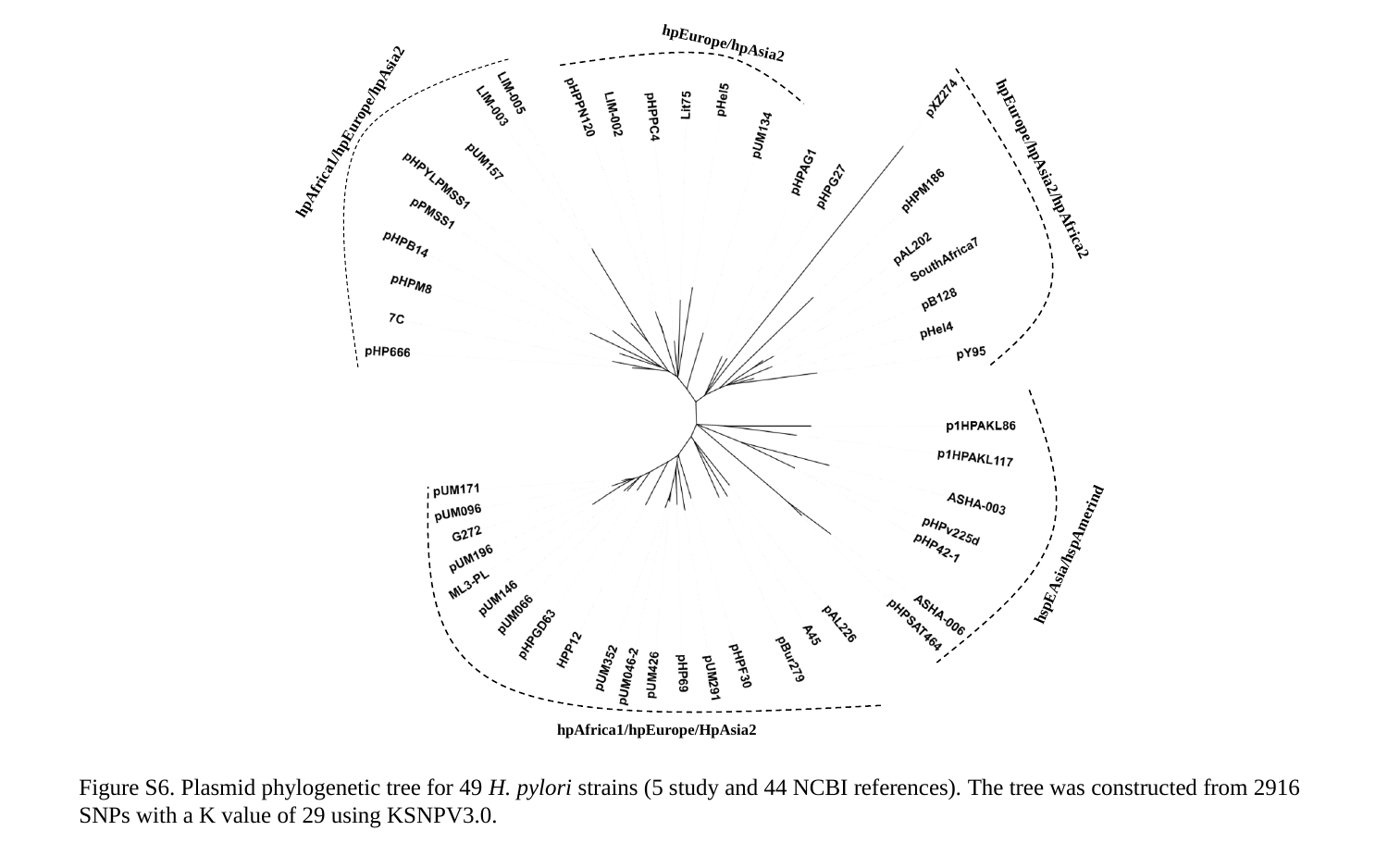

hpEurope/hpAsia2
hpAfrica1/hpEurope/hpAsia2
hpEurope/hpAsia2/hpAfrica2
hspEAsia/hspAmerind
hpAfrica1/hpEurope/HpAsia2
Figure S6. Plasmid phylogenetic tree for 49 H. pylori strains (5 study and 44 NCBI references). The tree was constructed from 2916 SNPs with a K value of 29 using KSNPV3.0.
